# Validation of the suction device Nimble for the assessment of skin fibrosis in systemic sclerosis

**DOI:** 10.1186/s13075-020-02214-y

**Published:** 2020-06-03

**Authors:** Bettina Müller, Lisa Ruby, Suzana Jordan, Marga B. Rominger, Edoardo Mazza, Oliver Distler

**Affiliations:** 1 Institute for Mechanical Systems, ETH Zurich, 8092 Zurich, Switzerland; 2 Institute of Diagnostic and Interventional Radiology, University Hospital Zurich, 8091 Zurich, Switzerland; 3 Department of Rheumatology, University Hospital Zurich, Gloriastrasse 25, 8091 Zurich, Switzerland; 4 Empa, Swiss Federal Laboratories for Materials Science and Technology, 8600 Dubendorf, Switzerland

**Keywords:** Systemic sclerosis, Suction measurement, Skin stiffness, Skin fibrosis

## Abstract

**Objectives:**

Skin fibrosis is a main hallmark of systemic sclerosis (SSc). Clinical assessment is done semi-quantitatively using the modified Rodnan skin score (mRSS). Objective measurements for quantifying skin fibrosis could complement the mRSS to achieve higher reproducibility. The aim of this study was to explore the potential of suction measurements to detect structural changes in the skin that are associated with skin fibrosis.

**Methods:**

This clinical trial included 30 SSc patients and 30 healthy volunteers (HC). We validated a novel suction device—the Nimble—to quantify skin stiffness in comparison to the Cutometer using the OMERACT filter.

**Results:**

A significant difference (*p* < 0.05) between the skin stiffness of HC and SSc patient groups was found for each location measured. The correlation between the measurements of forearm skin stiffness and the mRSS values was high for the Nimble (*r* = 0.82) and moderate for the Cutometer (*r* = 0.58). A ROC analysis showed good ability for the Nimble to distinguish between SSc patients with and without skin involvement (AUC = 0.82). Both suction devices provided excellent reliability in all measurements on HC and SSc patients and proved face validity and feasibility.

**Conclusion:**

Suction devices assessing skin stiffness, such as the Nimble, show clear potential to objectively quantify skin fibrosis in SSc patients and might be promising outcome measures complementing established methods such as the mRSS.

**Trial registration:**

Clinicaltrials.gov, NCT03644225, Registered 23 August 2018—Retrospectively registered, http://www.clinicaltrials.gov

## Introduction

Systemic sclerosis (SSc) is a heterogenic, autoimmune, connective tissue disease, characterized by vasculopathy and inflammation in the remodeling of tissue architecture [1]. Skin fibrosis is one main hallmark of SSc and common in many patients with the disease [2]. It has been shown that the severity of skin fibrosis [3, 4] and its rate of progression [5, 6] reflect the prevalence of internal organ involvement. According to ACR/EULAR 2013 criteria [7], skin thickening on the fingers that extends proximal to the metacarpophalangeal joints is sufficient for the classification of SSc. Unfortunately, diagnosing and evaluating the extent and activity of skin involvement is sometimes difficult for clinicians, due to the rarity of the disease and their limited experience with its symptoms [8]. Furthermore, assessment of skin fibrosis in SSc patients is affected by the low sensitivity and specificity of outcome measures. As a consequence, therapies for skin fibrosis could not be shown to be effective using common non-invasive quantification methods [1].

The modified Rodnan skin score (mRSS) is the gold standard for the clinical assessment of skin fibrosis in SSc patients. It is based on a palpation method that assesses skin thickness in combination with skin tethering at 17 sites on the body, with scores of 0 (normal) to 3 (severe) [9]. The mRSS is the most widely used measure for evaluating drug efficacy on skin fibrosis. Its applicability, however, has only been validated for the early disease stages of diffuse cutaneous SSc [10], and the drawback of this method is the high inter-observer variability of about 25% [11]. Nevertheless, the mRSS-based quantification method is the most common primary outcome measure used in clinical trials [12, 13]. It is also a major component of the composite outcome measure CRISS, which has been used in recent clinical trials [14]. Furthermore, the mRSS at baseline has been shown to predict worsening [15] or improvement [16] of skin fibrosis, thereby improving the definition of inclusion criteria for clinical trials. Taken together, the literature shows the utility of skin involvement measures for classifying and treating SSc. However, there is an urgent need for objective measures of skin fibrosis in order to improve the design and reliability of clinical trials.

An alternative approach for quantifying skin fibrosis is measuring the biomechanical properties of the tissue with the suction principle [17]. Characterization of skin mechanical properties based on the suction method has been shown to detect relevant differences in SSc patients [17, 18]. The most widely used suction device is the Cutometer, which has demonstrated valuable applicability for the quantification of skin involvement in SSc patients [19]. However, the measurement is prone to errors due to inter- and intra-observer variability [20] arising from observer and patient movements influencing the contact force [21–23]. These problems are associated with the large weight and size of the Cutometer probe. With this in mind, we developed a novel suction device with strongly reduced probe dimensions and weight [20]. We propose that this novel device is more effective in distinguishing fibrotic skin from healthy skin and therefore could be useful for assessing skin fibrosis in SSc patients.

In the present study, we aim to validate skin suction for use with SSc patients in accordance with the Outcome Measures in Rheumatology (OMERACT) filter. More specifically, the objective of this study is to assess the capabilities of two suction devices, the Nimble and the Cutometer, to quantify skin involvement in SSc patients in comparison with the mRSS, the current clinical gold standard.

## Materials and methods

### Patients and healthy controls

Patients scheduled for a yearly routine assessment at the University Hospital Zurich’s Department of Rheumatology were enrolled in this study. Inclusion criteria were fulfillment of ACR/EULAR 2013 criteria for the classification of SSc and the presence of skin fibrosis as assessed by the mRSS. Thirty age- and sex-matched healthy volunteers (HC) were enrolled as a control group.

An interventional medical device study (registered at www.clinicaltrials.gov: NCT03644225) was performed at the University Hospital Zurich in accordance with the approval obtained from the local ethics committee (Cantonal Ethics Committee Zurich, KEK-ZH-Nr. 2017-01154) and from the Swiss National Agency for Therapeutic Products (Swissmedic, 2017-MD-0045). Each patient and control signed a written informed consent form. The study was performed according to good clinical practice (GCP) guidelines, including external monitoring and compliance with the Declaration of Helsinki.

### Modified Rodnan skin score (mRSS)

Our study clinically assessed skin fibrosis with the mRSS by palpation according to the standardized method [12]. All 17 body sites selected for measurement were evaluated by trained clinicians. Suction measurements were performed at four specific body sites: the left and right dorsal forearms and the back of the left and right hands. This is why we define mRSS^4^_total_ as the sum of the mRSS values of these four locations:

$$ {\mathrm{mRSS}}_{\mathrm{total}}^4={\mathrm{mRSS}}_{\mathrm{back}\ \mathrm{of}\ \mathrm{left}\ \mathrm{hand}}+{\mathrm{mRSS}}_{\mathrm{back}\ \mathrm{of}\ \mathrm{right}\ \mathrm{hand}}+ $$

$$ +{\mathrm{mRSS}}_{\mathrm{left}\ \mathrm{dorsal}\ \mathrm{forearm}}+{\mathrm{mRSS}}_{\mathrm{right}\ \mathrm{dorsal}\ \mathrm{forearm}} $$

The total mRSS describing the sum of the scores of all 17 body sites will be referred to as mRSS^17^_total_.

### OMERACT filter

Data analysis was based on the OMERACT filter [24] and interpreted as follows: the face validity of the measurement methods was evaluated and explained using measurements on a controlled system (synthetic material) that mimicked skin fibrosis (stiffer material behavior). Feasibility was judged due to the duration of the measurement and patients’ tolerance for it. Content validity was interpreted as the ability of the outcome measures to distinguish between HC and SSc patients, and the ability to differentiate between severity grades of skin fibrosis. The correlation of the study outcome measure with the mRSS, the clinical gold standard for quantifying skin involvement in SSc patients, and the convergent validity based on the correlation of the stiffness measure of the Nimble (the novel device) with the stiffness measure of the Cutometer (the validated commercial device) was used to analyze criterion validity. Specificity and sensitivity of the new diagnostic method additionally proved criterion validity. Construct validity was assessed through comparison of the diagnostic measure with other related outcome measures like the mRSS^17^_total_ and lung fibrosis (high-resolution computed tomography, HRCT). The evaluation of intraclass correlations (reliability) was based on analysis of the intraclass correlation coefficient (ICC).

### Suction devices

**Fig. 1:**
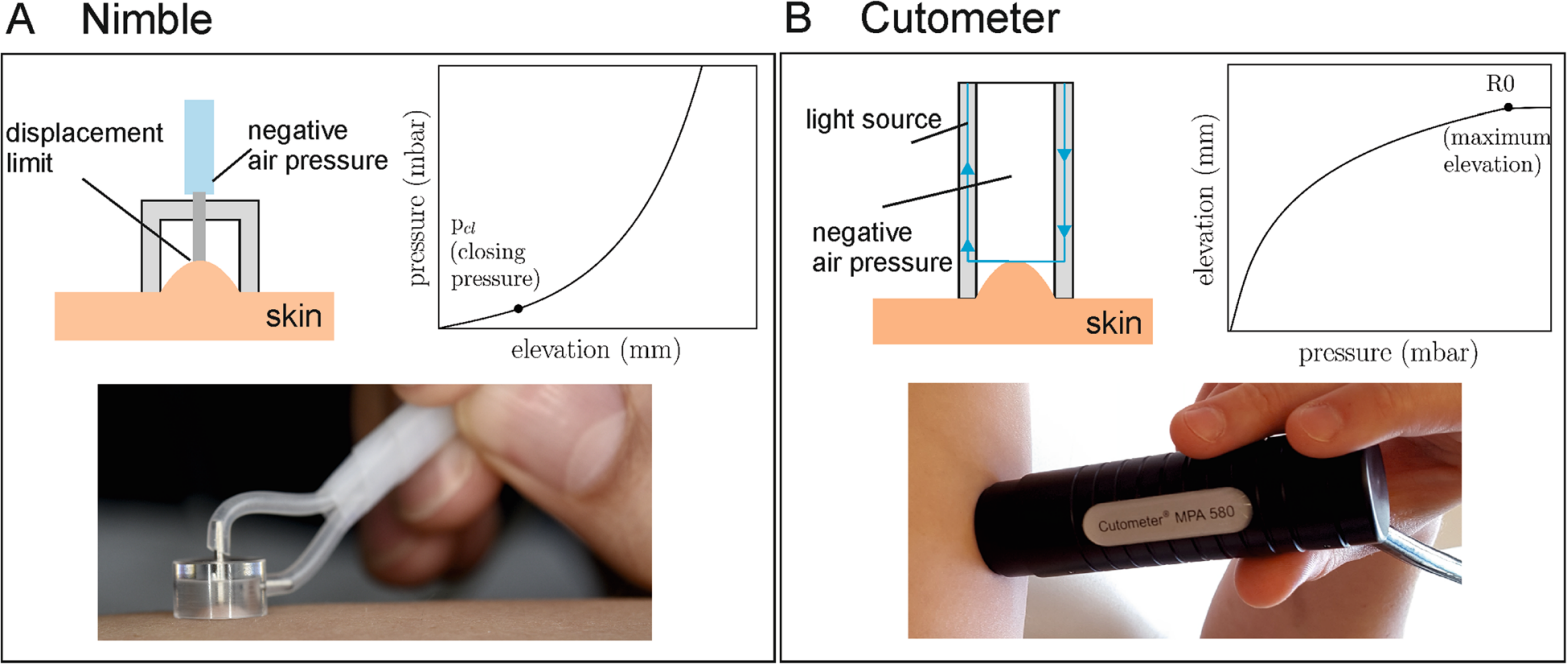
Schemes of the working principles of the Nimble (**a**) and Cutometer (**b**) suction devices. The Nimble operates in a displacement-controlled fashion, with negative pressure drawing the skin into the probe opening until it reaches a defined height (*h*). The outcome measure is the pressure (*p*_cl_) needed for the specific tissue elevation. The Cutometer operates in a load-controlled fashion, with negative pressure drawing the skin into the probe opening until a maximum pressure is reached. The outcome measure is the elevation corresponding to a specific suction load. We extracted the maximum elevation (R0 in mm) for our study

We first determined the mechanical parameters with two different devices (Fig. 1). The Cutometer MPA 580 (Courage & Khazaka electronic, Cologne, Germany) applies a defined negative pressure on the surface of the skin, drawing it into a circular opening measuring 6 mm in diameter. The elevation of the tissue is measured with an optical measurement system. Our suction tests applied what is called the Mode 2 protocol, using *p*_max_ = 250 mbar and a pressure ramp of 15 mbar/s. Importantly, based on Mueller et al. [20], we applied a correction scheme for each measurement with the Cutometer in order to minimize the influence of the initial contact force. The maximum tissue elevation is the outcome of the measurement (parameter R0_corr_ in mm).

The Nimble is a novel lightweight suction device that minimizes operator influence on the measurement outcome [20]. Negative pressure draws the skin into the 6-mm probe opening until it reaches a defined height (*h* = 0.5 mm). The pressure (*p*_cl_) needed for this specific elevation is determined in each test.

The stiffness parameters *k*^Nimble^ (Nimble) and *k*^R0^ (Cutometer) were calculated as the outcome measures of the suction devices:

$$ {k}^{\mathrm{R}0}=\frac{p_{\mathrm{max}}}{R{0}_{\mathrm{corr}}}=\frac{250\ \mathrm{mbar}}{R{0}_{\mathrm{corr}}};{k}^{\mathrm{Nimble}}=\frac{p_{\mathrm{cl}}}{h}=\frac{p_{\mathrm{cl}}}{0.5\ \mathrm{mm}} $$

In order to enable comparability with the mRSS, we express the suction results as the skin stiffness score *k*SS, which was calculated for each device as follows:

$$ {k\mathrm{SS}}_{\mathrm{loc}}=\frac{k_{\mathrm{loc}}^{\mathrm{SSc}}}{{\overline{k}}_{\mathrm{loc}}^{\mathrm{HC}}}; $$

$$ {k\mathrm{SS}}_{\mathrm{total}}={k\mathrm{SS}}_{\mathrm{back}\ \mathrm{of}\ \mathrm{left}\ \mathrm{hand}}+{k\mathrm{SS}}_{\mathrm{back}\ \mathrm{of}\ \mathrm{right}\ \mathrm{hand}}+ $$

$$ +{k\mathrm{SS}}_{\mathrm{left}\ \mathrm{dorsal}\ \mathrm{forearm}}+{k\mathrm{SS}}_{\mathrm{right}\ \mathrm{dorsal}\ \mathrm{forearm}} $$

*k*SS is the stiffness measured at a specific location (*k*_loc_) of an SSc patient and normalized with the average stiffness at the same location measured on the healthy controls (${\overline{k}}_{\mathrm{loc}}^{\mathrm{HC}}\Big)$. The *k*SS_total_ is the summation of the *k*SS over the four measured locations.

### Measurement procedure

One observer measured skin stiffness at four body sites, the left and right dorsal forearms and the back of the left and right hands, on a total of 30 SSc patients and 30 healthy controls. The observer began the measurement procedure with the Nimble device and measured each location four times in a row. The observer waited at least 35 s between repeated measurements at the same location. The total measurement duration for each location was approximately 5 min. Afterwards, the same procedure was repeated with the Cutometer device. We generated an outcome parameter—tissue stiffness—for each measurement. In order to apply the correction scheme on Cutometer outcomes, the pre-deformation of the initial contact force was recorded in each measurement. The location-specific stiffness *k*_loc_ was determined as the average of the four measurements.

### Statistical analysis

Statistical analysis was performed using the Python library scipy.stats (Python Software Foundation, Delaware, USA). The analysis included descriptive statistics, means, standard deviation (SD), and standard error of the mean (SEM). The ability of the devices to distinguish between HC and SSc patients was determined using a two-sided *t*-test (stats.ttest_ind) with a level of significance *p* < 0.05. The reliability of the devices in distinguishing between individual patients and healthy volunteers was calculated using the intraclass correlation coefficient ICC [1, 2], which is based on a random single measurement. We used the ICC categorization of Cicchetti [25], where ICC = 0.4 shows poor reliability of the outcome measure, 0.4 < ICC < 0.59 is fair, 0.6 < ICC < 0.74 is good, and 0.75 < ICC < 1.0 is excellent. Concurrent validity was assessed between the stiffness measures *k*^Nimble^ and *k*^R0^ from the suction experiments, as well as between the stiffness measures and the mRSS of the clinical assessment. To this end, Pearson’s correlation (stats.pearsonr) was used with the following interpretation [26]: 0.00 < *r* < 0.35 indicates weak correlation, 0.36 < *r* < 0.67 moderate correlation, and 0.68 < *r* < 1.0 high correlation between the outcome measures. An area under curve (AUC) of receiver operating characteristic (ROC) analysis was performed to evaluate the discrimination between SSc with mRSS^4^_total_ = 0 and mRSS^4^_total_ > 0 of the stiffness parameters *k*^Nimble^ and *k*^R0^. Youden’s index [27] was used to evaluate the most suitable cut-off value.

## Results

### Demographics of SSc patients and HCs

The characteristics and demographics of the 30 SSc patients included in the trial are summarized in Table 1. The 30 HCs were 55 ± 10.4 years old, and 23 out of 30 were female (76.7%).

**Table 1:** Demographics and clinical characteristics of SSc patients (*n* = 30) at baseline. Definitions of items and organ manifestation are according to EUSTAR [28]. Data is presented as number (*n*)/total valid cases (*N*) (%). Disease duration was calculated as the difference between the date of the baseline visit and the date of the first non-Raynaud’s symptom of the disease as reported by the patient. Pulmonary hypertension was judged on RHC after application of the DETECT score. Active disease was defined as a score > 3 by calculating European Scleroderma Study Group disease activity indices for systemic sclerosis as proposed by Valentini [29]. Immunosuppressive therapy was defined as treatment with corticosteroids (prednisone dose ≥ 10 mg/day or other dosage forms in equal dose) or any immunosuppressant. *ACA* anti-centromere antibody, *ANA* antinuclear antibody, *Anti-Scl-70* anti-topoisomerase antibody, *CRP* C-reactive protein, *HRCT* high-resolution computed tomography, *FVC* forced vital capacity, *mRSS* modified Rodnan skin score, *NYHA* New York Heart Association, *VAI* Valentini Activity Index

Characteristics	SSc patients
**Demographics**	
Age (mean ± SD)	58.3 ± 11.1
Sex: female	21/30 (70%)
Disease duration (mean ± SD, in years)	11.1 ± 7.9
ACR/EULAR criteria fulfilled	30/30 (100%)
**Subtype**	
Diffuse SSc	8/30 (26.7%)
**Skin/vascular**	
Raynaud’s phenomenon	29/30 (96.7%)
Digital ulcers (past and present)	19/30 (63.3%)
Current digital ulcers	7/28 (25%)
Pitting scars	19/29 (65.5%)
Scleredema	24/28 (85.7%)
Telangiectasia	18/30 (60%)
mRSS (mean ± SD)	7.2 ± 7.1
Abnormal nailfold capillaroscopy	26/26 (100%)
**Musculoskeletal**	
Tendon friction rubs	0
Joint synovitis	3/28 (10.7%)
Joint contractures	11/27 (40.7%)
Muscle weakness	3/28 (10.7%)
**Gastrointestinal**	
Esophageal symptoms	17/30 (56.6%)
Stomach symptoms	13/30 (43.3%)
Intestinal symptoms	16/30 (53.3%)
**Cardiopulmonary**	
Dyspnea	
NYHA Stage 1	11/29 (37.9%)
NYHA Stage 2	14/29 (48.3%)
NYHA Stage 3/4	4/29 (13.8%)
Diastolic dysfunction	7/28 (25%)
Conduction blocks	2/28 (7.1%)
PAH by RHC	2/28 (7.1%)
Lung fibrosis on HRCT	15/29 (51.7%)
FVC, % predicted (mean ± SD)	92.1 ± 17.5
FVC < 70% predicted	2/28 (7.1%)
**Kidney**	
Renal crisis	0
**Laboratory parameters**	
ANA positive	30/30 (100%)
ACA positive	12/28 (42.8%)
Anti-Scl-70 positive	9/30 (30%)
Anti-RNA-polymerase III positive	8/28 (28.6%)
CRP elevation	5/27 (18.5%)
Active disease (VAI > 3)	5/26 (19.2%)
Immunosuppressive therapy	7/30 (23.3%)

We evaluated the mean and SEM of the mRSS quantification from the clinical assessment for SSc patients for the four different body sites that were evaluated in this study (Supplementary Fig. 1). The values for the back of the right hand were $\overline{{\mathrm{mRSS}}_{\mathrm{BHR}}}=0.73\pm 0.17$, for the left $\overline{{\mathrm{mRSS}}_{\mathrm{BHL}}}=0.67\pm 0.16$, and for the dorsal forearms $\overline{{\mathrm{mRSS}}_{\mathrm{DVR}}}=0.43\pm 0.14$ and $\overline{{\mathrm{mRSS}}_{\mathrm{DVL}}}=0.33\pm 0.12$.

### Face validity and feasibility

**Fig. 2:**
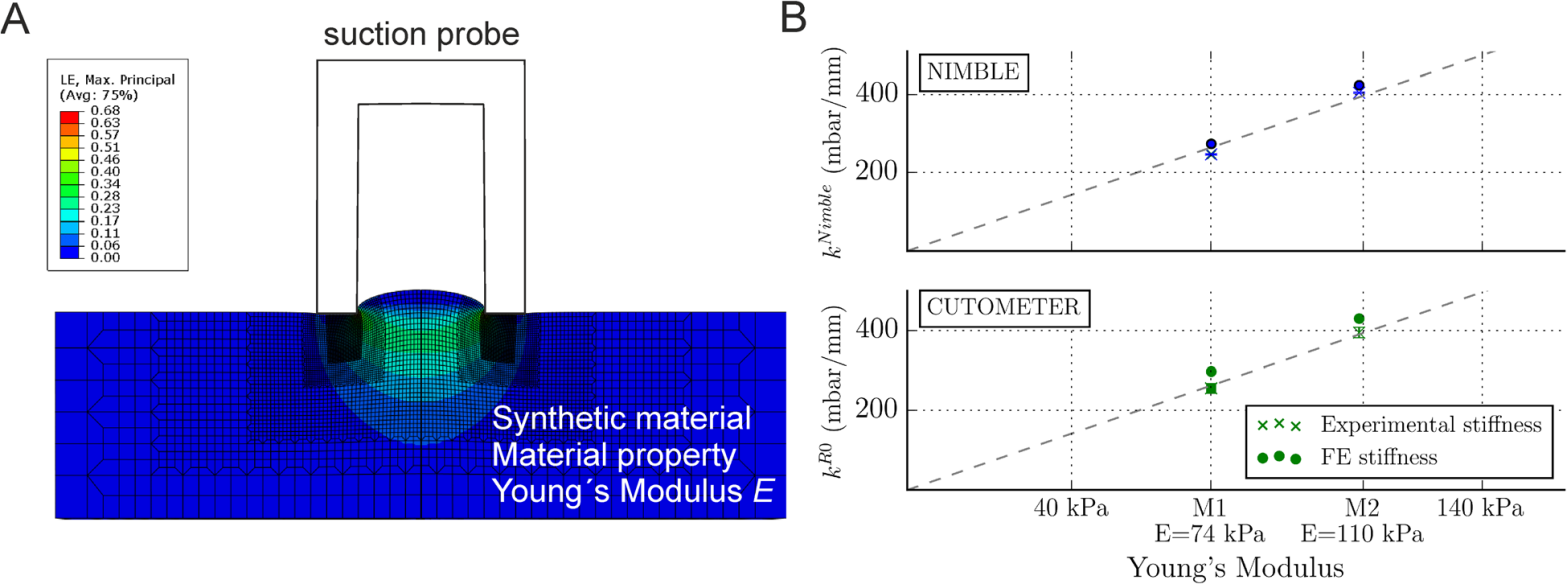
**a** Finite element (FE) simulation of suction experiment using a neo-Hookean material model with Young’s modulus E. The 2D cross-sectional geometry is depicted. **b** Estimated *k*^Nimble^ and *k*^R0^ for two materials, M1 and M2, with FE analysis (FE stiffness) and mean (*n* = 3) of *k*^Nimble^ and *k*^R0^ (experimental stiffness). The dotted line indicates the linear relationship between *k*^Nimble^ or *k*^R0^ and the Young’s modulus of the material

We tested the face validity of the suction procedure on a synthetic material. The advantage of using a synthetic material is the control one has over mechanical properties such as Young’s modulus (*E*), which describes the stiffness of a material, i.e., the relationship between the applied force and the deformation it generates. We manufactured two compliant elastomers characterized by a stiffness corresponding to soft skin (*E*_*M1*_ = 74 kPa) and stiff skin (*E*_*M2*_ = 110 kPa*).* We then measured *k*^Nimble^ and *k*^R0^ (Fig. 2b) and performed corresponding numerical simulations of suction, i.e., finite element (FE) analyses using a specific constitutive model (neo-Hookean) with corresponding material parameters (Fig. 2a). As shown in Fig. 2b, the stiffness of Material 1 (M1) was *k*^Nimble^_M1_ = 246.52 ± 1.69 mbar/mm and *k*^R0^_M1_ = 255.46 ± 12.3 mbar/mm (mean and SD), and for Material 2 (M2), the stiffness was *k*^Nimble^_M2_ = 404.99 ± 1.33 mbar/mm and *k*^R0^_M2_ = 394.77 ± 13.32 mbar/mm. These suction measurements were very much in line with the computational predictions of the FE analysis (*k*^FE-Nimble^_M1_ = 274.10 mbar/mm, *k*^FE-Nimble^_M2_ = 424.10 mbar/mm, and *k*^FE-R0^_M1_ = 297.62 mbar/mm, *k*^FE-R0^_M2_ = 430.29 mbar/mm). The dashed lines indicate the relationship between the actual stiffness of the material (Young’s modulus) and the measured stiffness (*k*^Nimble^ and *k*^R0^), which followed a linear proportionality. Thus, suction devices can differentiate between the stiffness of materials and have face validity to assess tissue stiffness in skin fibrosis.

Feasibility was given, as the procedure itself took less than 5 min per body site and the measurements were very well tolerated by the patients without any reported adverse event.

### Content validity (comprehensiveness) of stiffness measures

**Fig. 3:**
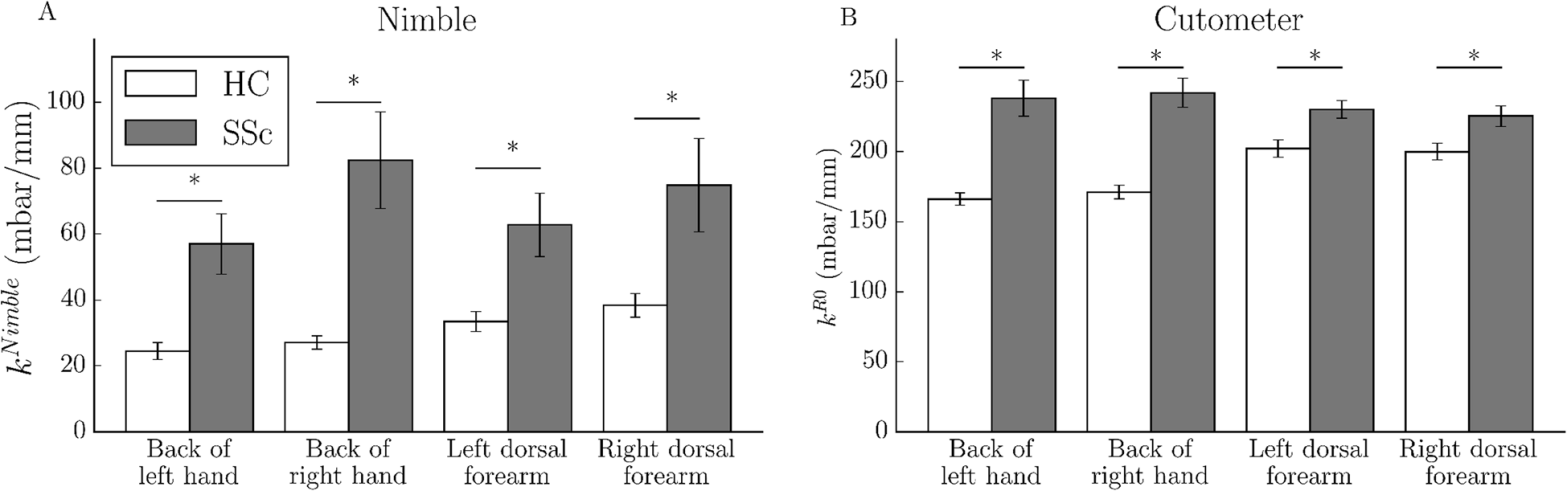
Mean and SEM for HC (white) and SSc (gray) measured with the Nimble (**a**) and the Cutometer (**b**) for the four body locations, *n* = 30 for each group. For all locations and both devices, significant differences were found with a paired *t*-test (*p* < 0.0)

Content validity was assessed by investigating the ability of the suction method to distinguish between HC and SSc patients. Figure 3 depicts the mean and SEM of the stiffness measure for each location of HC (white) and SSc patients (gray) measured with the Nimble (Fig. 3a) and the Cutometer (Fig. 3b). The average coefficient of variation was larger for the Nimble (19.7%) compared to the Cutometer (6.8%). Significant differences (*p* < 0.05) between HC and SSc patients were found for each location and for both devices.

**Fig. 4:**
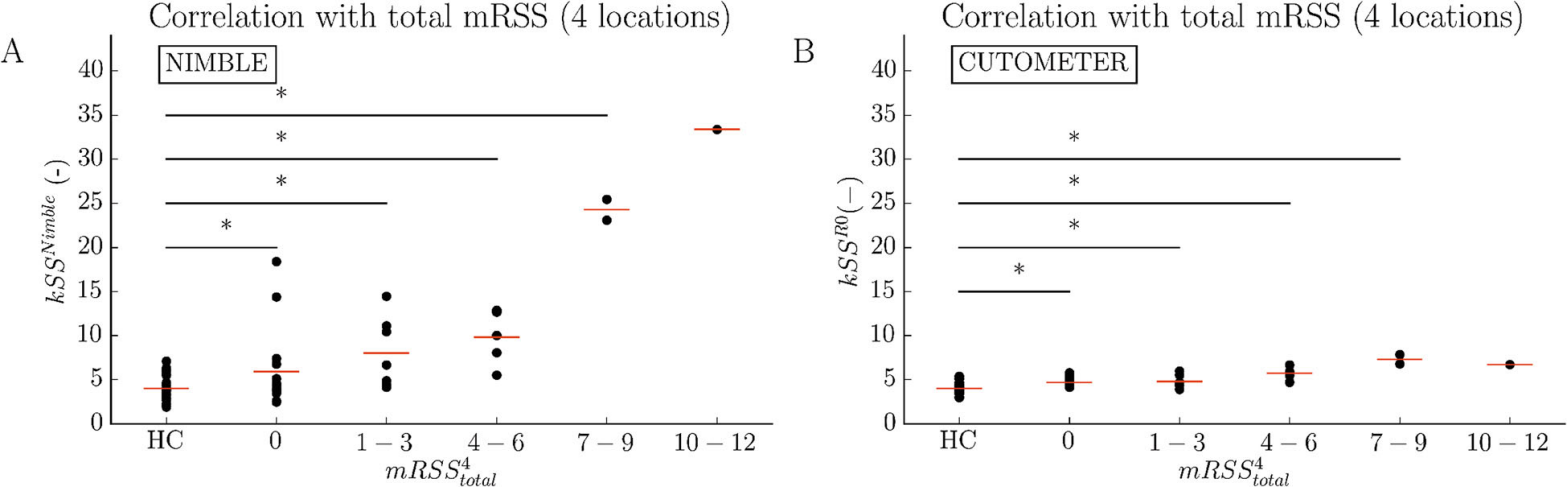
Skin stiffness score of suction measurements correlated with mRSS values of SSc patients. **a** Correlation of *k*SS^Nimble^ with mRSS^4^_total_ of the four measured locations. The first group includes the *k*SS^Nimble^ for the HC group, the second the *k*SS^Nimble^ for the SSc patients with total mRSS^4^_total_ = 0 and the following grouped in mRSS ranges of: 1 ≤ mRSS^4^_total_ ≤ 3, 4 ≤ mRSS^4^_total_ ≤ 6, 7 ≤ mRSS^4^_total_ ≤ 9 and 10 ≤ mRSS^4^_total_ ≤ 12. **b** Correlation of kSS^R0^ with mRSS^4^_total_ of the four measured locations

Content validity was further assessed by examining whether the suction devices can differentiate between severity grades of skin fibrosis. Figure 4 shows the skin stiffness scores *k*SS^Nimble^ (Fig. 4a) and *k*SS^R0^ (Fig. 4c) with grouped mRSS^4^_total_ values of the four measured locations. The stiffness score increased with higher mRSS^4^_total_ values suggesting good content validity. We found significant differences between the *k*SS^Nimble^ and *k*SS^R0^ of the HC group and SSc patients with mRSS^4^_total_ = 0, mRSS^4^_total_ between 1 and 3, 4 and 6, and 7 and 9.

### Criterion validity

Criterion validity can be assessed by comparing the performance with the gold standard, e.g., the mRSS. We analyzed Pearson’s correlation coefficient *r* between mRSS and *k*^Nimble^ or *k*^R0^, respectively, as well as *r* between the stiffness measures *k*^R0^ and *k*^Nimble^ (Supplementary Table 1). Correlations between mRSS and *k*^Nimble^ were found to be moderate (*r* = 0.47 and *r* = 0.57) for measurements on the back of the hand and high (*r* = 0.82 and *r* = 0.74) for measurements on the dorsal forearms, respectively. Correlations with *k*^R0^ were moderate for all locations (*r* = 0.62, *r* = 0.56 on the back of the hands, and *r* = 0.58 on the dorsal forearms). When comparing the stiffness measures *k*^Nimble^ and *k*^R0^, we observed high correlations for the back of the left and right hands as well as for the right dorsal forearm (*r* = 0.82, *r* = 0.81 and *r* = 0.71) and a moderate correlation for measurements of the left dorsal forearm (*r* = 0.64), respectively. These data indicate a location-dependent moderate to good criterion validity of the suction devices.

**Fig. 5:**
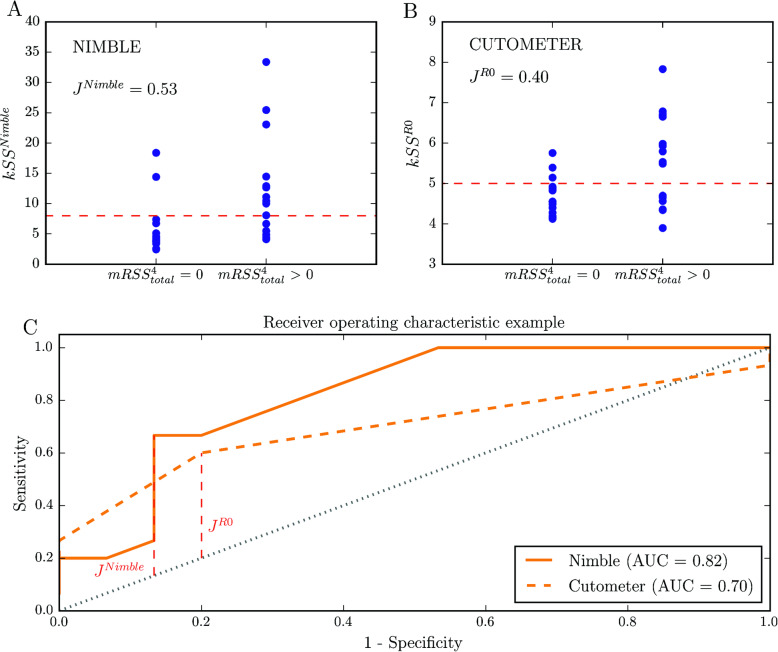
**a** Skin stiffness score (*k*SS) of Nimble measurements grouped into SSc patients with mRSS^4^_total_ = 0 and mRSS^4^_total_ > 0. **b** Skin stiffness score (*k*SS) of Cutometer measurements grouped into SSc patients with mRSS^4^_total_ = 0 and mRSS^4^_total_ > 0. **c** Receiver operating characteristic (ROC) curve tests for sensitivity and specificity of suction measures. Nimble measurements showed a larger area under the ROC curve (AUC) compared to Cutometer measurements, indicating a better ability to distinguish between SSc patients with a total mRSS^4^_total_ = 0 and SSc patients with a total mRSS^4^_total_ > 0. Cut-off values were evaluated by the Youden’s index, indicated in red

Another aspect of criterion validity is the specificity and sensitivity of the new diagnostic method. To address this point, we grouped the SSc patients into two groups: the first group with a total mRSS^4^_total_ = 0 and the second with mRSS^4^_total_ > 0. Figure 5 a and b show *k*SS^Nimble^ and *k*SS^R0^ for the two groups. Based on this data, we performed an ROC analysis and evaluated the most suitable cut-off value to differentiate between normal and fibrotic skin using a calculation of the Youden’s index *J*. For the outcome measure of the Nimble, we found a higher *J*^Nimble^ = 0.53 compared to *J*^R0^ = 0.40 and cut-off values at *k*SS^Nimble^ = 8 and *k*SS^Cutometer^ = 5, respectively. ROC analysis of the accuracy of the stiffness measure (Fig. 5c) revealed that discrimination between SSc patients with an mRSS^4^_total_ = 0 and mRSS^4^_total_ > 0 was better for the Nimble (*AUC* = 0.82) than for the Cutometer (*AUC* = 0.70).

### Construct validity

Construct validity can be assessed by comparing the diagnostic measure of interest with other related outcome measures. We analyzed the correlation of suction measurements with other fibrotic disease measures such as lung fibrosis measured by high-resolution computed tomography (HRCT) (Supplementary Fig. 2) and the total mRSS^17^_total_ of the 17 locations (Supplementary Fig. 3). *k*SS^Nimble^ measures were grouped into SSc patients with and without lung fibrosis, and the mean value was *k*SS^Nimble^ = 11.54 mbar/mm for patients with lung fibrosis and *k*SS^Nimble^ = 6.32 mbar/mm for those without (*p* = 0.063). The same analysis was performed for Cutometer stiffness outcomes and mRSS^17^_total_. The following mean values were observed: *k*SS^R0^ = 5.57 mbar/mm with lung fibrosis and *k*SS^R0^ = 4.61 mbar/mm without (*p* < 0.05), and mRSS^17^_total_ = 8.87 for patients with lung fibrosis detected by HRCT and mRSS^17^_total_ = 5.57 for those without (*p* = 0.22). For the total mRSS^17^_total_ of all 17 body locations, we found high Pearson’s correlations with the skin stiffness score *k*SS^Nimble^ (*r =* 0.73) and *k*SS^R0^ (*r =* 0.66), shown in Supplementary Table 1. Additionally, linear regression analysis showed increasing skin stiffness with increasing mRSS^17^_total_ (Supplementary Fig. 3). The obtained regression values were as follows: *R*^*2*^*(k*SS^Nimble^*)* = 0.547 and *R*^2^(*k*SS^R0^) = 0.268.

### Reliability

The reproducibility of the suction devices was tested by measuring the intraclass correlation coefficient (ICC). The ICC estimates the ability of the outcome measure to distinguish between patients and healthy controls for four repeated measurements at each location. We found excellent ICC values (ICC^Nimble^ = 0.76 and ICC^R0^ = 0.81) for the HC group and even higher ICC values (ICC^Nimble^ = 0.91 and ICC^R0^ = 0.85) for SSc patients. Note that very high ICC values were obtained with Nimble despite its larger coefficient of variation.

## Discussion

One of the main characteristics of SSc is skin fibrosis, a condition which is marked by the massive deposition of collagen fibers in the extracellular matrix. As a consequence, the skin tissue becomes stiffer and thicker and experiences tethering to the underlying tissue. With this study, we aimed to evaluate two suction devices, the Nimble and the Cutometer, with regard to their diagnostic potential for skin fibrosis in SSc patients. Our results confirm (Fig. 3) the ability of both suction devices to distinguish between tissue stiffness in HC and in SSc patients for all sites on the body that were measured.

The suction method provides an objective alternative to the mRSS [17], as it induces a deformation similar to lifting the skin between the fingers [19]. Our measurements showed a high correlation between *k*^Nimble^ and mRSS for measurements on the dorsal forearm (Supplementary Table 1). The moderate correlation between suction outcomes and mRSS for measurements on the back of the hand might be associated with the influence of the anatomical features under the skin. The presence of bones, tendons, and vessels leads to large variability between adjacent locations on the back of each patient’s hand. Additionally, slight tilting of fingers or the hand can form wrinkles, which interfere with suction measurements, leading to potential misinterpretation of the actual skin stiffness. As there are several studies (e.g., [17, 30]) proving the validity of the Cutometer, we interpret the strong correlation between the two suction devices as an additional confirmation of criterion validity for the Nimble. The present study applies a correction scheme previously developed for the Cutometer in order to minimize the influence of the variable contact force exerted during the measurements [20]. It is important to note that data analysis without this correction leads to much worse performance and higher variability of Cutometer measurements: the ICC values of *k*^R0^ fall to ICC^R0^ = 0.69 for HC and ICC^R0^ = 0.75 for SSc patients, and the area under the curve in ROC analysis drops to AUC = 0.66.

The value of the newly designed Nimble device in detecting fibrotic skin was shown to be superior to that of the Cutometer. The results in Fig. 5 could be considered confirmation of the ability of the Nimble to predict whether the patient’s skin is clinically involved. Additionally, suction outcomes were found to correlate with the total mRSS^17^_total_ of all 17 body locations and even showed associations with other fibrotic disease measures (Supplementary Fig. 2). Even though we only compared the *kSS* values of four body sites with the overall disease measures of SSc, conformity could be observed. Intraclass correlation coefficients indicated excellent reliability for the suction method for both HC and SSc patients.

In skin fibrosis, healthy extracellular matrix is replaced with collagen-rich connective tissue [31]. This profuse collagen deposition results in stiffer tissue behavior [1]. This condition is currently indirectly quantified by the mRSS method, which mainly reflects the perceived skin thickness. However, tissue stiffness is predominantly determined by the density and condition of collagen fibers, and this does not necessarily correlate with tissue thickness. Based on the present results, we propose that measuring tissue stiffness with the suction approach is a more appropriate way of quantifying skin fibrosis than relying on the mRSS alone. In Fig. 2, we showed the ability of suction measurements to accurately quantify the stiffness of synthetic materials with different stiffness properties. We expect a suitable suction procedure to be able to quantify tissue stiffness due to higher collagen deposition. Such a method, however, would not be able to distinguish between stiffness resulting from higher collagen density and stiffness resulting from stretched collagen fibers due to edema.

The novel device (Nimble) was shown to provide a promising alternative to the existing suction device (Cutometer). It is easy to use and inherently safe, and the low costs of the Nimble probe allow it to be used as a disposable device adding to its feasibility. The Nimble’s measurement duration was the same as for the Cutometer: for four repeated measurements, it amounted to 5 min per measured location.

The main limitation of this study is the rather small sample size when considering the heterogeneous clinical presentation of SSc. The objective was to analyze the validity of suction measurements for detecting structural skin changes in SSc patients. While this was confirmed over the wide range of the present patient cohort, the number of patients with high mRSS values was rather low. The present study also did not address sensitivity to change over time: no longitudinal measurements were performed, and the discrimination capacity over the course of treatment was not evaluated. Similarly, due to the small sample size, it was not possible to evaluate the influence of disease duration on biomechanical parameters.

## Conclusion

The diagnostic relevance of biomechanical measurements was analyzed in a clinical trial involving 30 SSc patients. The results of the present study fulfill the OMERACT filter [32], including *face validity*, *content validity*, *criterion validity*, *construct validity*, *reliability*, and *feasibility* of suction as an objective measurement procedure for skin involvement in SSc patients. Our results are in line with biomechanical measurements for other fibrotic diseases [33–35]. The reliability and feasibility of suction measurements suggest that this method could be a promising complement for clinical assessment of skin fibrosis in patients with SSc.

## Supplementary information

**Additional file 1. **Mean and SEM of mRSS quantification from clinical assessment of SSc patients for the four measured locations, *n* = 30.

**Additional file 2.** Pearson’s correlation coefficient r for evaluation of criterion and construct validity for both suction devices. Pearson’s correlation between stiffness measures k^Nimble^ and k^R0^ with mRSS (moderate to high) indicates criterion validity. Construct validity is assessed by Pearson’s correlation between k^Nimble^ and k^R0^.

**Additional file 3.** (A) Skin stiffness score (kSS) of Nimble measurements grouped into SSc patients with no lung fibrosis on HRCT and presence of lung fibrosis on HRCT. The horizontal line indicates the mean of kSS^Nimble^. (B) Skin stiffness score (kSS) of Cutometer measurements grouped into SSc patients with no lung fibrosis on HRCT and presence of lung fibrosis on HRCT. The horizontal line indicates the mean of kSS^R0^. (C) Total mRSS^17^_total_ of 17 locations grouped into SSc patients with no lung fibrosis on HRCT and presence of lung fibrosis on HRCT. The horizontal line indicates the mean of mRSS^17^_total_.

**Additional file 4.** A) Linear regression (R^2^) of kSS^Nimble^ with mRSS^17^_total_ of 17 body locations. The regression line assumes kSS^Nimble^ = 4 for mRSS^17^_total_ = 0. The slope m of the regression line is indicated. (B) Linear regression of kSS^R0^ with mRSS^17^_total_ of the 17 body locations.

## Data Availability

The datasets used and analyzed during the current study are available from the corresponding author on reasonable request.

## Abbreviations

ACA: Anti-centromere antibody
ACR: American College of Rheumatology
ANA: Antinuclear antibody
Anti-Scl-70: Anti-topoisomerase antibody
AUC: Area under curve
CRISS: Composite response index in dcSSc
CRP: C-reactive protein
EULAR: European League against Rheumatism
EUSTAR: European Scleroderma Trials and Research group
FE: Finite element
FVC: Forced vital capacity
GCP: Good clinical practice
HC: Healthy volunteers
HRCT: High-resolution computed tomography
ICC: Intraclass correlation coefficient
mRSS: Modified Rodnan skin score
NYHA: New York Heart Association
OMERACT: Outcome Measures in Rheumatology
PAH: Pulmonary arterial hypertension
RHC: Right heart catheterization
ROC: Receiver operating characteristic
SD: Standard deviation
SEM: Standard error of the mean
SSc: Systemic sclerosis
VAI: Valentini Activity Index

## References

[1] Allanore Y, Simms R, Distler O, Trojanowska M, Pope J, Denton CP (2015). Systemic sclerosis. Nat Rev Dis Prim.

[2] Khanna D, Merkel PA (2007). Outcome measures in systemic sclerosis: an update on instruments and current research. Curr Rheumatol Rep.

[3] Shand L, Lunt M, Nihtyanova S, Hoseini M, Silman A, Black CM (2007). Relationship between change in skin score and disease outcome in diffuse cutaneous systemic sclerosis: application of a latent linear trajectory model. Arthritis Rheum.

[4] Zheng B, Nevskaya T, Baxter CA, Ramey DR, Pope JE, Baron M, et al. Changes in skin score in early diffuse cutaneous systemic sclerosis are associated with changes in global disease severity. Rheumatology. 2019;59(2):398–406. 10.1093/rheumatology/kez299 31359048

[5] Domsic RT, Rodriguez-Reyna T, Lucas M, Fertig N, Medsger TA (2011). Skin thickness progression rate: a predictor of mortality and early internal organ involvement in diffuse scleroderma. Ann Rheum Dis.

[6] Wu W, Jordan S, Graf N, de Oliveira PJ, Curram J, Allanore Y (2019). Progressive skin fibrosis is associated with a decline in lung function and worse survival in patients with diffuse cutaneous systemic sclerosis in the European Scleroderma Trials and Research (EUSTAR) cohort. Ann Rheum Dis.

[7] van den Hoogen F, Khanna D, Fransen J, Johnson SR, Baron M, Tyndall A (2013). 2013 classification criteria for systemic sclerosis: an American College of Rheumatology/European League Against Rheumatism Collaborative Initiative. Arthritis Rheum.

[8] Denton CP, Khanna D (2017). Systemic sclerosis. Lancet..

[9] Clements PJ, Lachenbruch PA, Seibold JR, Zee B, Steen VD, Brennan P (1993). Skin thickness score in systemic sclerosis: an assessment of interobserver variability in 3 independent studies. J Rheumatol.

[10] Kumánovics G, Péntek M, Bae S, Opris D, Khanna D, Furst DE (2017). Assessment of skin involvement in systemic sclerosis. Rheumatology.

[11] Clements P, Lachenbruch P, Siebold J, White B, Weiner S, Martin R (1995). Inter and intraobserver variability of total skin thickness score (modified Rodnan TSS) in systemic sclerosis. J Rheumatol.

[12] Khanna D, Furst DE, Clements PJ, Allanore Y, Baron M, Czirjak L (2017). Standardization of the modified Rodnan skin score for use in clinical trials of systemic sclerosis. J Scleroderma Relat Disord.

[13] Distler O, Pope J, Denton C, Allanore Y, Matucci-Cerinic M, de Oliveira PJ (2017). RISE-SSc: Riociguat in diffuse cutaneous systemic sclerosis. Respir Med.

[14] Khanna D, Berrocal VJ, Giannini EH, Seibold JR, Merkel PA, Mayes MD (2016). The American College of Rheumatology Provisional Composite Response Index for clinical trials in early diffuse cutaneous systemic sclerosis. Arthritis Rheumatol.

[15] Maurer B, Graf N, Michel BA, Müller-Ladner U, Czirják L, Denton CP (2015). Prediction of worsening of skin fibrosis in patients with diffuse cutaneous systemic sclerosis using the EUSTAR database. Ann Rheum Dis.

[16] Dobrota R, Maurer B, Graf N, Jordan S, Mihai C, Kowal-Bielecka O (2016). Prediction of improvement in skin fibrosis in diffuse cutaneous systemic sclerosis: a EUSTAR analysis. Ann Rheum Dis.

[17] Dobrev HP (1999). In vivo study of skin mechanical properties in patients with systemic sclerosis. J Am Acad Dermatol.

[18] Nikkels-Tassoudji N, Henry F, Pierard-Franchimont C, Pierard GE (1996). Computerized evaluation of skin stiffening in scleroderma. Eur J Clin Investig.

[19] Enomoto DN, Mekkes JR, Bossuyt PM, Hoekzema R, Bos JD (1996). Quantification of cutaneous sclerosis with a skin elasticity meter in patients with generalized scleroderma. J Am Acad Dermatol.

[20] Müller B, Elrod J, Pensalfini M, Hopf R, Distler O, Schiestl C (2018). A novel ultra-light suction device for mechanical characterization of skin. Jan Y-K, editor. PLoS One.

[21] Bonaparte JP, Ellis D, Chung J (2013). The effect of probe to skin contact force on Cutometer MPA 580 measurements. J Med Eng Technol.

[22] Piérard GE, Nikkels-Tassoudji N, Piérard-Franchimont C (1995). Influence of the test area on the mechanical properties of skin. Dermatology..

[23] Hashmi F, Wright C, Nester C, Lam S (2015). The reliability of non-invasive biophysical outcome measures for evaluating normal and hyperkeratotic foot skin. J Foot Ankle Res.

[24] Kowal-Bielecka O, Avouac J, Pittrow D, Huscher D, Behrens F, Denton CP (2010). Echocardiography as an outcome measure in scleroderma-related pulmonary arterial hypertension: a systematic literature analysis by the EPOSS group. J Rheumatol.

[25] Cicchetti DV (1994). Guidelines, criteria, and rules of thumb for evaluating normed and standardized assessment instruments in psychology. Psychol Assess.

[26] Taylor R (1990). Interpretation of the correlation coefficient: a basic review. J Diagnostic Med Sonogr.

[27] Hughes G (2015). Youden’s index and the weight of evidence. Methods Inf Med.

[28] Meier FMP, Frommer KW, Dinser R, Walker UA, Czirjak L, Denton CP (2012). Update on the profile of the EUSTAR cohort: an analysis of the EULAR Scleroderma Trials and Research group database. Ann Rheum Dis.

[29] Valentini G (2001). European multicentre study to define disease activity criteria for systemic sclerosis. II. Identification of disease activity variables and development of preliminary activity indexes. Ann Rheum Dis.

[30] Dobrev H (2007). In vivo study of skin mechanical properties in Raynaud’s phenomenon. Skin Res Technol.

[31] Bhattacharyya S, Wei J, Varga J (2012). Understanding fibrosis in systemic sclerosis: shifting paradigms, emerging opportunities. Nat Rev Rheumatol.

[32] Kowal-Bielecka O, Avouac J, Pittrow D, Huscher D, Behrens F, Denton CP (2011). Analysis of the validation status of quality of life and functional disability measures in pulmonary arterial hypertension related to systemic sclerosis: results of a systematic literature analysis by the expert panel on outcomes measures in pulmonary art. J Rheumatol.

[33] Elrod J, Müller B, Mohr C, Meuli M, Mazza E, Schiestl C (2019). An effective procedure for skin stiffness measurement to improve Paediatric burn care. Burns..

[34] Lee KC, Bamford A, Gardiner F, Agovino A, ter Horst B, Bishop J (2020). Burns objective scar scale (BOSS): validation of an objective measurement devices based burn scar scale panel. Burns..

[35] Busche MN, Thraen A-CJ, Gohritz A, Rennekampff H-O, Vogt PM (2018). Burn scar evaluation using the Cutometer® MPA 580 in comparison to “Patient and Observer Scar Assessment Scale” and “Vancouver Scar Scale”. J Burn Care Res.

