# Supplementary figures and images for: Validation of the suction device Nimble for the assessment of skin fibrosis in systemic sclerosis
Source: Arthritis Res Ther. 2020 Jun 3;22:128. doi: 10.1186/s13075-020-02214-y (PMC7268280; doi:10.1186/s13075-020-02214-y)

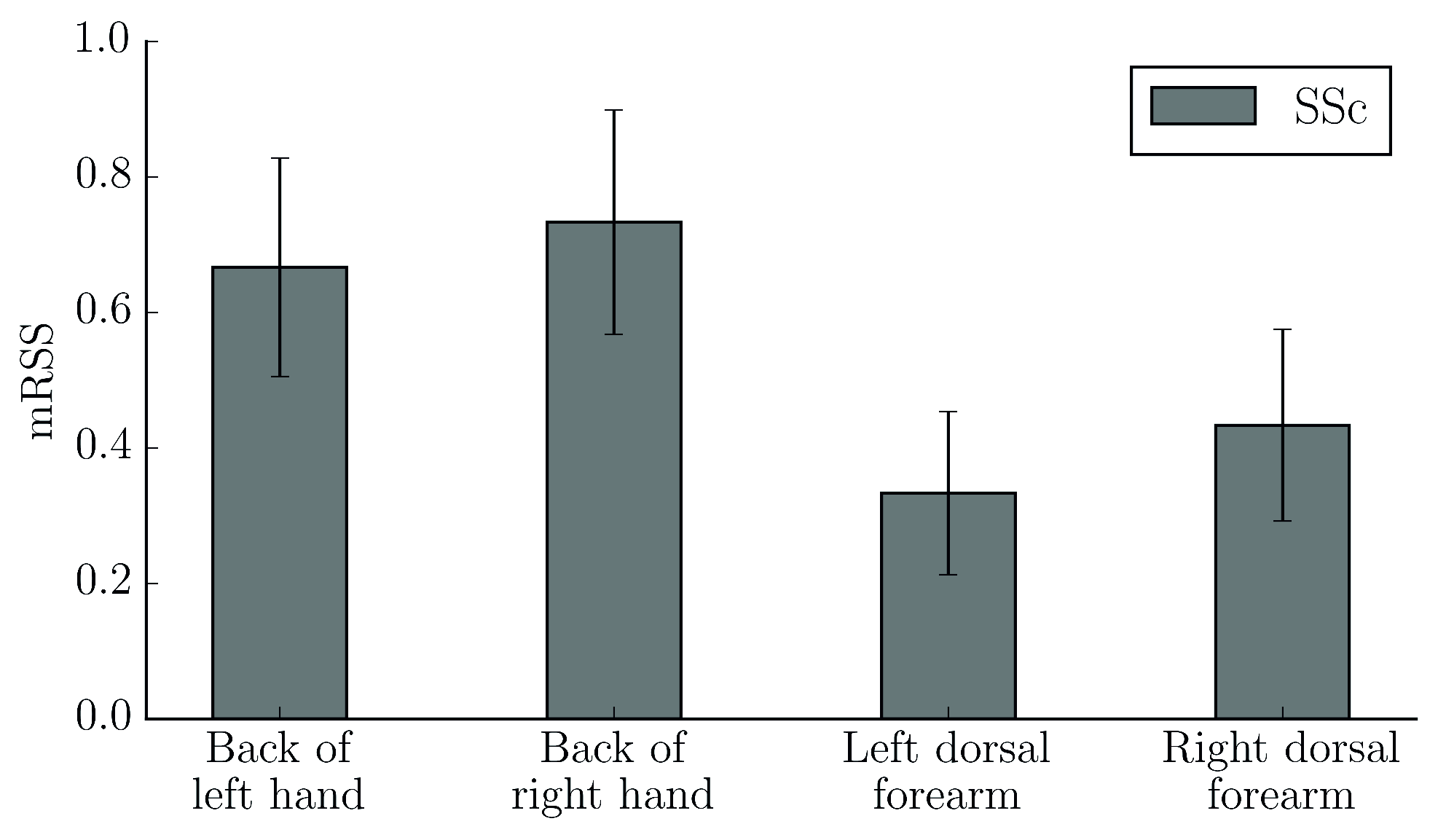

Supplement: Supplementary file 1 — Additional file 1. Mean and SEM of mRSS quantification from clinical assessment of SSc patients for the four measured locations, n = 30. [file 13075_2020_2214_MOESM1_ESM.tif]

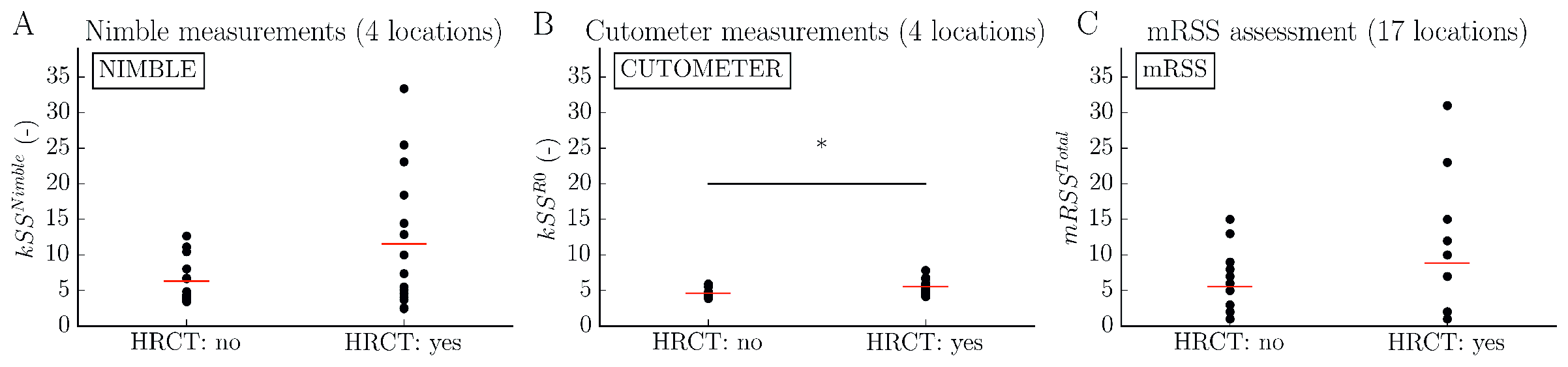

Supplement: Supplementary file 3 — Additional file 3. (A) Skin stiffness score (kSS) of Nimble measurements grouped into SSc patients with no lung fibrosis on HRCT and presence of lung fibrosis on HRCT. The horizontal line indicates the mean of kSSNimble. (B) Skin stiffness score (kSS) of Cutometer measurements grouped into SSc patients with no lung fibrosis on HRCT and presence of lung fibrosis on HRCT. The horizontal line indicates the mean of kSSR0. (C) Total mRSS17total of 17 locations grouped into SSc patients with no lung fibrosis on HRCT and presence of lung fibrosis on HRCT. The horizontal line indicates the mean of mRSS17total. [file 13075_2020_2214_MOESM3_ESM.tif]

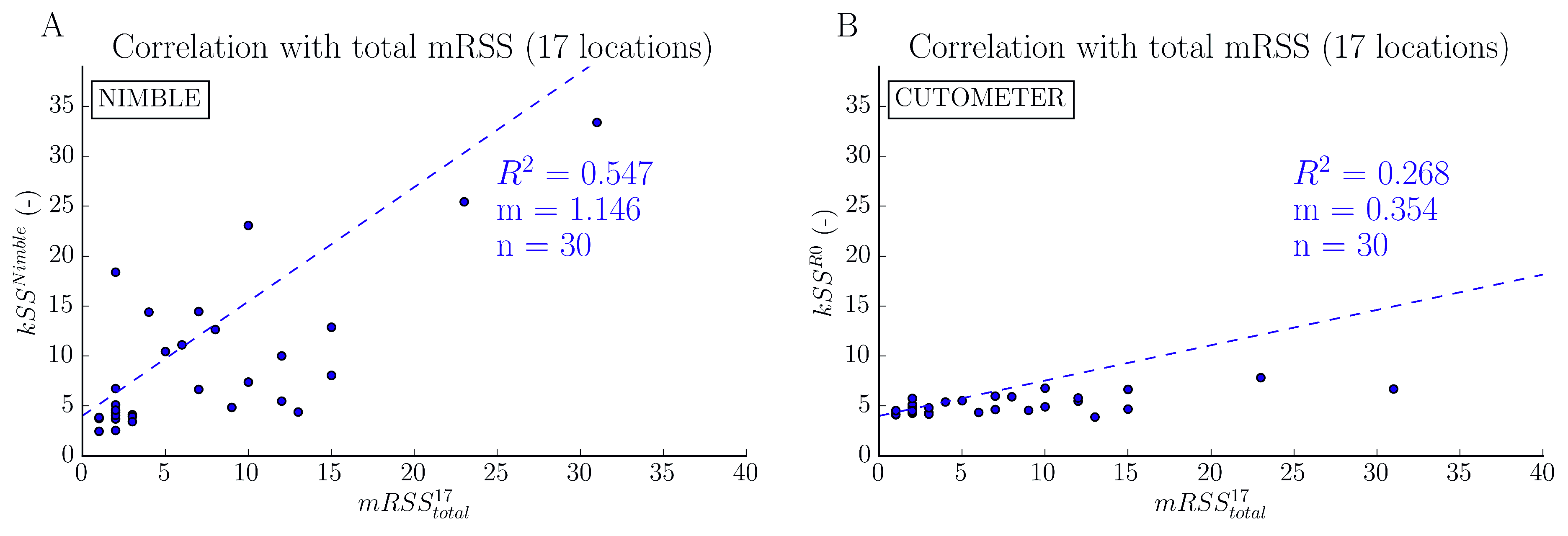

Supplement: Supplementary file 4 — Additional file 4. A) Linear regression (R2) of kSSNimble with mRSS17total of 17 body locations. The regression line assumes kSSNimble = 4 for mRSS17total = 0. The slope m of the regression line is indicated. (B) Linear regression of kSSR0 with mRSS17total of the 17 body locations. [file 13075_2020_2214_MOESM4_ESM.tif]
