# Supplementary material for: Validation of the suction device Nimble for the assessment of skin fibrosis in systemic sclerosis
Source: Arthritis Res Ther. 2020 Jun 3;22:128. doi: 10.1186/s13075-020-02214-y (PMC7268280; doi:10.1186/s13075-020-02214-y)
Supplement: Supplementary file 2 — Additional file 2. Pearson’s correlation coefficient r for evaluation of criterion and construct validity for both suction devices. Pearson’s correlation between stiffness measures kNimble and kR0 with mRSS (moderate to high) indicates criterion validity. Construct validity is assessed by Pearson’s correlation between kNimble and kR0. [file 13075_2020_2214_MOESM2_ESM.pdf]

	mRSS - k^{Nimble}	mRSS - k^{R0}	k^{R0} - k^{Nimble}
**Back of left hand**	0.47	0.62	**0.82**
**Back of right hand**	0.57	0.56	**0.81**
**Left dorsal forearm**	**0.82**	0.58	0.64
**Right dorsal forearm**	**0.74**	0.58	**0.71**
